# Selective transcript–protein discordance of glycolytic remodeling in human hypertrophic cardiomyopathy

**DOI:** 10.3389/fcvm.2026.1937619

**Published:** 2026-10-21

**Authors:** Jingyuan Chen, Die Hu, Yang Yang, Mingqi Ouyang, Xiaoman Chen

**Affiliations:** 1 Department of Cardiovascular Medicine, The Second Xiangya Hospital of Central South University, Changsha, Hunan, China; 2 Department of Cardiovascular Medicine, Guilin Hospital of the Second Xiangya Hospital, Central South University, Guilin, China

**Keywords:** glycolysis, HIF1α, hypertrophic cardiomyopathy, metabolic remodeling, multi-omics, phosphoproteomics, transcriptome-proteome decoupling

## Abstract

**Background:**

Although metabolic reprogramming has attracted growing attention in hypertrophic cardiomyopathy (HCM), transcriptomic and proteomic studies have yielded conflicting views on glycolytic alterations. As a master transcriptional regulator of glycolytic genes, HIF1α remains poorly characterized in human HCM tissue.

**Methods:**

We integrated four transcriptomic datasets, three proteomic datasets, and one phosphoproteomic dataset from publicly available human HCM cohorts. Glycolysis-, hypoxia-, and oxidative metabolism-related pathways were assessed by gene set enrichment analysis (GSEA). Transcript-protein concordance was evaluated by comparing the direction and magnitude of individual glycolytic enzyme changes across omics layers.

**Results:**

Across all four transcriptomic cohorts, glycolysis-related programs were suppressed at the mRNA level (NES range: −1.1 to −1.7). HALLMARK_HYPOXIA was negatively enriched in four of five transcriptomic comparisons, and Semenza HIF1 targets showed the strongest suppression (NES = −1.99, *P*adj = 2.8 × 10^−4^). At the protein level, alterations differed by pathway. Oxidative pathways (OXPHOS, TCA cycle, FAO) showed concordant downregulation at RNA and protein levels, whereas glycolytic enzymes displayed level-specific discordance. In Garmany 2023 paired samples, 5 of 12 glycolytic enzymes showed opposite RNA and protein directions, whereas the overall RNA–protein correlation was weak and non-significant (*r* = −0.28, *P* = 0.38; *n* = 12) and was therefore considered descriptive. HK1 was the most prominent example of transcript-proteome decoupling, with reduced transcripts in four transcriptomic cohorts but increased protein levels across three independent proteomic platforms (Garmany TMT, Nollet LC-MS/MS, Lumish SomaScan). GSEA in Garmany 2023 demonstrated opposite enrichment patterns between transcriptome and proteome for Glycolysis (NES −1.11 vs. +1.40) and Hypoxia (NES −1.89, *P*adj < 0.001 vs. +1.34, ns), although proteomic enrichment did not reach FDR significance. Phosphoproteomic analysis revealed enzyme-specific rather than pathway-wide changes: HK1 (log2FC + 0.18, FDR < 10^−5^) and PGAM1 (+0.40, FDR = 0.02) showed increased phosphorylation, whereas ALDOA, LDHB, PKM, and PFKM showed decreased phosphorylation (all FDR < 0.01).

**Conclusions:**

Our study identifies a transcript-protein disconnect in glycolytic metabolism in human HCM. HIF1-associated transcriptional programs were reduced rather than activated. Oxidative pathways (OXPHOS, TCA cycle, FAO) showed coordinated downregulation across RNA and protein levels, whereas glycolysis exhibited frequent RNA-protein divergence, suggesting distinct regulatory mechanisms across metabolic pathways in HCM.

## 1 Introduction

Hypertrophic cardiomyopathy (HCM) is the most prevalent heritable heart disease, found in roughly 1 in 500 people, and is defined pathologically by asymmetric thickening of the left ventricle, disorganized myocyte architecture, and interstitial fibrosis (1, 2). Sarcomere gene mutations—particularly in MYH7 and MYBPC3—explain about half of cases, yet the chain of events from a mutant sarcomere to a clinical phenotype remains only partly charted (3). Among the secondary processes that contribute to disease progression, metabolic remodeling stands out as an increasingly important link between sarcomere dysfunction, energy depletion, and clinical worsening (4, 5).

Under normal conditions, the adult heart generates roughly 60%–70% of its ATP through mitochondrial fatty acid oxidation (FAO); glucose oxidation, ketone bodies, and amino acids make up the balance (6). When the heart hypertrophies pathologically, FAO capacity drops and glycolysis ramps up—a so-called “metabolic switch” that has been documented in numerous animal models of pressure overload (7, 8). In those models, HIF1α acts as a key transcriptional activator, driving expression of glycolytic enzymes, glucose transporters, and lactate dehydrogenase (9, 10). PET-FDG imaging in HCM patients does show elevated myocardial glucose uptake, which at first glance fits neatly into this framework (11, 12).

But the picture gets murkier when one moves beyond imaging to direct tissue measurements. Multi-omics profiling of human HCM myectomy specimens has uncovered deep deficits in FAO enzymes and acylcarnitines, and—more puzzlingly—contradictory shifts among glycolytic pathway members (13, 14). Nollet et al. (15), for instance, found that glycolytic enzyme proteins were actually more abundant in HCM tissue even though lactate levels fell—an observation hard to reconcile with uniformly elevated glycolytic flux (15). Two questions emerge from these conflicting observations. Do the protein-level changes match what is happening at the mRNA level? And are HIF1-associated transcriptional programs truly switched on in human HCM, as the animal literature would predict? Neither question has been addressed systematically across multiple human cohorts.

At its core, this is a question about whether mRNA faithfully predicts protein. Discordance between transcript and protein abundance is well recognized in biology—arising from translational regulation, mRNA stability differences, or protein turnover—but what it means for cardiac metabolism has barely been explored (16, 17). In HCM, where energy supply-demand mismatch sits at the heart of the disease, the answer matters a great deal: if transcriptional data do not reliably reflect the functional proteome, then conclusions drawn from RNA-only studies could be misleading, and therapeutic strategies built on those conclusions may miss the mark.

We set out to address these gaps by assembling eight independent datasets spanning four transcriptomic cohorts (two snRNA-seq, one microarray, one bulk RNA-seq), three proteomic platforms (TMT, LC-MS/MS, SomaScan), and one phosphoproteomic dataset. The goals were straightforward: (1) test whether glycolytic transcript suppression is reproducible across cohorts; (2) compare, gene by gene, the direction of change at the transcript vs. protein level for glycolytic enzymes; (3) assess HIF1-associated transcriptional programs using curated target gene sets; and (4) look for phosphorylation signatures that might indicate post-translational compensation. What we found was unexpected—a glycolysis-specific disconnect between transcription and protein abundance, combined with reduced HIF1 associated transcriptional programs—and it forces a reconsideration of how metabolic remodeling actually operates in human HCM.

## 2 Methods

### 2.1 Datasets

The following publicly available datasets were included in this study:

Transcriptomic datasets: (1) Chaffin et al. 2022 (Nature) (14): single-nucleus RNA-sequencing (snRNA-seq) of cardiac tissue from 16 non-failing (NF) donors, 8 HCM with preserved ejection fraction (HCMpEF), and 7 HCM with reduced ejection fraction (HCMrEF), totaling 420,693 nuclei profiled on the 10× Genomics platform (SCP1303). (2) Liu et al. 2023 (Cell Discovery) (18): snRNA-seq from 3 control and 10 HCM hearts comprising 52,640 nuclei (figshare 5777948). (3) GSE36961 (19): Illumina bead-array microarray profiling of 106 HCM and 39 control septal myectomy and donor heart samples, representing the largest single microarray cohort in HCM. (4) Garmany et al. 2023 (Circulation: Genomic and Precision Medicine) (13): bulk RNA-seq of flash-frozen myectomy samples from 97 HCM patients and 23 controls, with paired proteomic and phosphoproteomic data.

Proteomic datasets: (1) Garmany et al. (13): TMT-based mass spectrometry proteomics on a representative subset of the RNA-seq cohort, enabling direct paired transcriptome-proteome comparison. (2) Nollet et al. 2024 (Circulation: Genomic and Precision Medicine) (15): LC-MS/MS-based proteomics and targeted metabolomics of 47 HCMpEF myectomy samples and 20 NF donor tissues (PXD046187). (3) Lumish et al. 2024 (JACC) (20): SomaScan-based plasma and tissue proteomics measuring 7,289 proteins in HCM vs. NF cardiac tissue.

Phosphoproteomic dataset: Garmany et al. (13): TMT-based phosphoproteomics performed on the same myectomy cohort, enabling assessment of post-translational modification changes in metabolic enzymes.

### 2.2 Single-nucleus RNA-seq analysis

For the Chaffin (14) and Liu (18) datasets, published quality-controlled cell annotations were retained. Raw counts were summed within each donor and cell type to generate donor-level pseudobulk count matrices. For the Chaffin dataset, Cell Ranger raw counts were used for pseudobulk aggregation. Genes with non-zero counts in fewer than three donors were excluded before differential-expression analysis. DESeq2 was applied directly to raw pseudobulk counts, with library-size normalization performed through DESeq2 size-factor estimation. HCMpEF vs. NF and HCMrEF vs. NF were analyzed separately in the Chaffin cohort, whereas HCM vs. control was analyzed in the Liu cohort. The primary Chaffin model included disease group only (∼HCM_type). To assess potential confounding by sex, the Chaffin analyses were repeated as a sensitivity analysis using sex + HCM_type. Wald statistics were used for gene ranking in subsequent GSEA.

snRNA-seq dropout sensitivity analysis. To quantify transcript sparsity, the fraction of cardiomyocyte nuclei with non-zero Cell Ranger raw counts was calculated for each of the 12 core glycolytic genes within each donor. Detection fractions were summarized across NF, HCMpEF, and HCMrEF donors. To assess whether low transcript detection systematically biased effect estimates, Chaffin cardiomyocyte pseudobulk log2 fold changes were compared with transcript-level log2 fold changes from the independent Garmany bulk RNA-seq cohort. Spearman correlation was used to test the relationship between nucleus-level detection fraction and the absolute difference in log2 fold change between the snRNA-seq and bulk RNA-seq datasets. The analysis was additionally repeated after exclusion of the extreme low-detection outlier ALDOA.

### 2.3 Microarray analysis (GSE36961)

Processed expression values for GSE36961 (19) were obtained from the GEO Series Matrix using GEOquery. The dataset was generated using the Illumina HumanHT-12 V3.0 expression beadchip (GPL15389); the deposited expression values had been Fastlo-normalized and log2-transformed by the original investigators, and no additional between-array normalization was applied. Differential expression between HCM and control samples was assessed using limma with empirical Bayes moderation. Probe-to-gene annotation was obtained from the platform annotation, and when multiple probes mapped to the same gene, the probe with the highest mean expression was retained.

### 2.4 Bulk RNA-seq and proteomic analysis (Garmany 2023)

For the Garmany cohort (13), published processed differential-analysis results were used. Bulk RNA-seq log2 fold changes and *P* values were obtained from the original edgeR analysis of 97 HCM and 23 control myocardial samples. TMT proteomic and phosphoproteomic log2 fold changes and multiple-testing-adjusted *P* values were obtained from the processed supplementary results of the original study. These published statistics were used for gene-level cross-omics comparisons and pathway ranking; differential-abundance models were not refitted from raw mass-spectrometry spectra in the present study. Each dataset was analyzed independently, and no cross-study expression matrix was constructed; therefore, no cross-cohort batch correction was applied. Integration across cohorts was performed only at the level of gene-level effect estimates and pathway enrichment statistics. Potential residual technical and cohort-specific heterogeneity was considered when interpreting cross-dataset comparisons. For the cross-proteomic comparison, published processed results were used and no differential-abundance model was refitted. For Nollet 2024, protein-level fold changes and nominal *P* values were taken from the Proteomics sheet of the original Supplementary Table. Differential analysis in the original study used limma on DIA-NN/MaxLFQ intensities. For Lumish 2024, fold changes and nominal *P* values were extracted from Supplementary Table. Lumish protein names were mapped to gene symbols by protein name, and only proteins with an unambiguous match to a single gene were retained. Fold changes were expressed as log2 values (signed linear fold changes in the Nollet table were converted as sign × log2|FC|). In Figure 5B, dot color indicates log2 fold change (HCM vs. non-failing donors; capped at ±2) and dot size indicates −log10 (nominal P) (capped at 2); small grey dots indicate *P* ≥ 0.10. Because the Lumish Supplementary Table S1 lists only proteins with nominal *P* < 0.05 and provides no protein-level FDR, both datasets are displayed using nominal *P* values, and this comparison is descriptive.

### 2.5 Gene set enrichment analysis

GSEA was performed using the fgsea package with 10,000 permutations and a fixed random seed [set.seed(42)]. Gene ranking was dataset-specific. For the Chaffin 2022 and Liu 2023 snRNA-seq analyses, genes were ranked by the DESeq2 Wald statistic. For GSE36961, genes were ranked by the moderated t-statistic from limma. For the Garmany 2023 bulk RNA-seq and proteomic analyses, genes or proteins were ranked by the published log2 fold-change values from the processed edgeR and TMT proteomic results, respectively. The following gene set collections were used: MSigDB Hallmark collection (H), including HALLMARK_GLYCOLYSIS, HALLMARK_HYPOXIA, HALLMARK_OXIDATIVE_PHOSPHORYLATION, HALLMARK_FATTY_ACID_METABOLISM, and HALLMARK_MTORC1_SIGNALING; Reactome pathways (C2:CP:REACTOME), including REACTOME_CITRIC_ACID_CYCLE_TCA_CYCLE, REACTOME_FATTY_ACID_METABOLISM, and REACTOME_AEROBIC_RESPIRATION_AND_RESPIRATORY_ELECTRON_TRANSPORT; curated HIF1α target gene sets from MSigDB C2 collection: SEMENZA_HIF1_TARGETS, ELVIDGE_HIF1A_TARGETS_UP, and PID_HIF1A_PATHWAY. Pathway enrichment was considered significant at FDR < 0.05.

### 2.6 Three-layer glucose metabolism analysis

Glucose metabolism genes were stratified into three functional layers: Layer 1 (Glucose Uptake): SLC2A1, SLC2A3, SLC2A4; Layer 2 (Glycolysis Core Enzymes): HK1, HK2, PFKL, PFKM, ALDOA, GAPDH, PGK1, PGAM1, ENO1, PKM, LDHA, LDHB; Layer 3 (Pyruvate Oxidation/TCA Coupling): PDK4, PDHA1, MPC1, MPC2, CS, IDH2, OGDH, SUCLA2, FH, MDH2. Log2 fold changes and FDR-adjusted *P*-values were extracted for each gene across all datasets and visualized as a dot plot with bubble size proportional to significance.

### 2.7 Transcriptome-proteome decoupling analysis

For the Garmany 2023 paired dataset (13), transcriptomic log2FC values were directly compared with proteomic log2FC values for 12 core glycolytic enzymes. Concordance was defined as same-direction changes at both levels; discordance as opposite-direction changes. Pearson correlation was computed between RNA and protein log2FC values. For pathway-level comparison, GSEA was performed independently on transcriptomic and proteomic ranked lists using the same gene sets. For cross-dataset validation, Chaffin 2022 pseudobulk transcriptomic log2FC (HCMpEF vs. NF) (14) was compared with Nollet 2024 proteomic log2FC (15), as both datasets represent HCMpEF (LVEF ≥ 55%) samples.

### 2.8 HIF1α target gene analysis

Three curated HIF1α target gene sets were assessed: SEMENZA_HIF1_TARGETS, ELVIDGE_HIF1A_TARGETS_UP, and PID_HIF1A_PATHWAY. GSEA was performed independently on transcriptomic and proteomic ranked lists from Garmany 2023. Consistency of HIF1α target gene suppression was evaluated across all five transcriptomic comparisons (four datasets).

### 2.9 Phosphoproteomic analysis

Differentially phosphorylated sites for glycolytic enzymes were extracted from Garmany 2023 phosphoproteomic data. Phosphoproteomic summary values provided in the Garmany dataset were used for the transcript–protein–phosphoprotein comparison. Site-resolved phosphopeptide data were subsequently examined to contextualize the potential functional consequences of selected phosphorylation changes. Because phosphorylation effects are residue-specific, phosphoproteomic abundance changes were not interpreted as direct measures of enzyme activity. FDR < 0.05 was considered significant. Transcriptomic, proteomic, and phosphoproteomic log2FC values were displayed in parallel for each glycolytic gene.

### 2.10 Statistical analysis

All statistical analyses were performed in R (version 4.3). For GSEA, Benjamini–Hochberg adjusted *P*-values were used with the same thresholds. Pearson correlation was used for transcriptome-proteome concordance assessment. Visualizations were generated using ggplot2, ggrepel, and patchwork packages.

## 3 Results

### 3.1 Glycolytic transcriptional programs are consistently reduced across independent HCM cohorts

We began by asking a simple question: is glycolytic gene expression consistently altered in HCM? To answer this, we ran GSEA across all four transcriptomic datasets. In the GSE36961 microarray cohort (19), the largest single dataset with 106 HCM samples, HALLMARK_GLYCOLYSIS showed significant negative enrichment (NES = −1.56, FDR < 0.001), and HALLMARK_HYPOXIA was even more strongly suppressed (NES = −1.91, FDR < 0.001) (Figure 1A). At the individual gene level, five of twelve core glycolytic enzymes were significantly downregulated (FDR < 0.05), with LDHA (log2FC = −0.94, FDR < 0.001), ENO1 (log2FC = −0.82, FDR < 0.001), and HK2 (log2FC = −0.35, FDR < 0.01) showing the largest reductions. Two enzymes, LDHB (log2FC = +0.20, FDR < 0.01) and PFKM (log2FC = +0.34, FDR < 0.01), were significantly upregulated, while the remaining five (PKM, PGK1, PGAM1, PFKL, GAPDH) showed no statistically significant change (Figure 1B).

**Figure 1:**
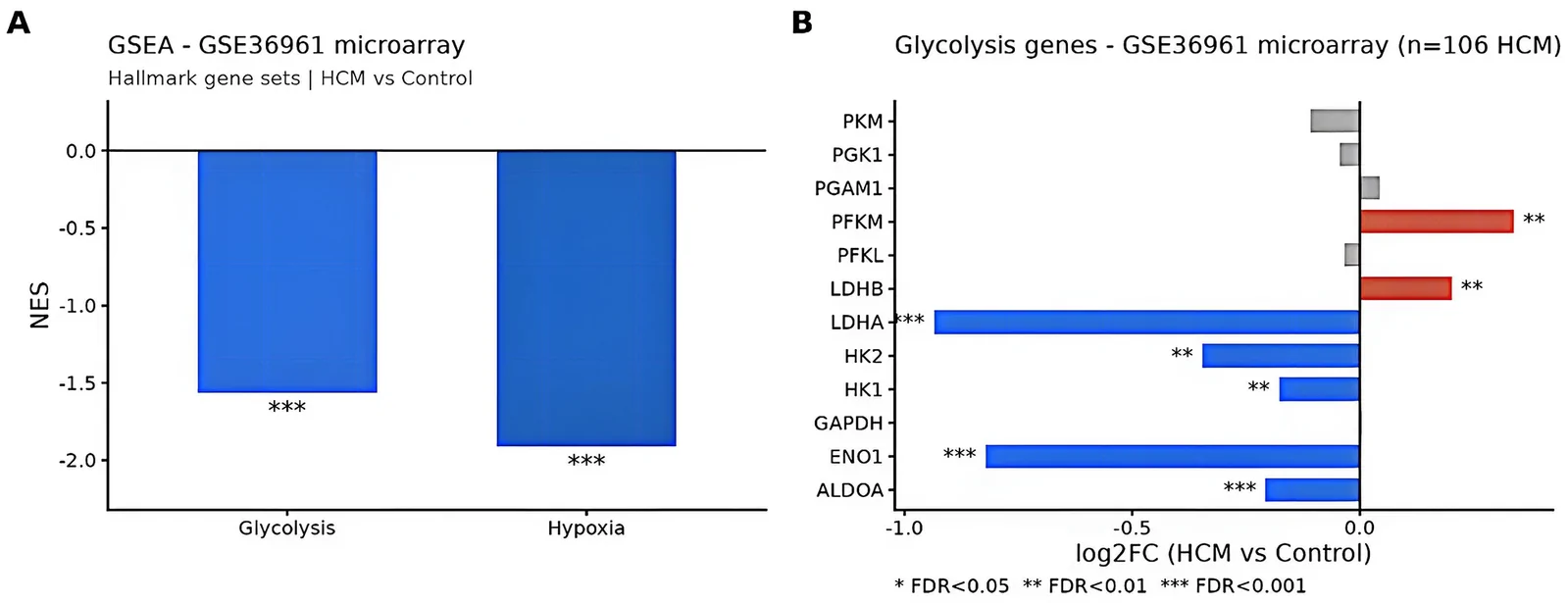
Suppression of glycolytic transcriptional programs in GSE36961 microarray cohort. **(A)** Gene set enrichment analysis (GSEA) of Hallmark gene sets in the GSE36961 microarray cohort. **(B)** Individual expression changes of 12 core glycolytic enzymes. Significance thresholds are based on adjusted *P*-values: *P < 0.05, **P < 0.01, ***P < 0.001. Differential expression was assessed using limma with empirical Bayes moderation.

Detection of the 12 core glycolytic transcripts in Chaffin cardiomyocyte nuclei varied substantially across genes. HK1, PFKM, PKM, and LDHB were detected in approximately 20%–44% of nuclei at the donor median, whereas HK2, PFKL, PGAM1, and LDHA showed considerably lower detection. ALDOA was an extreme low-detection outlier, with only 10 non-zero nuclei among 122,362 cardiomyocyte nuclei. Nevertheless, all other 11 glycolytic genes were detectable in every donor after pseudobulk aggregation. Chaffin cardiomyocyte log2 fold changes showed overall positive concordance with the independent Garmany bulk RNA-seq estimates (*r* = 0.79 for HCMpEF and *r* = 0.61 for HCMrEF). Importantly, lower nucleus-level detection was not associated with greater divergence from bulk RNA-seq effect estimates (Spearman *ρ* = 0.15 and 0.04 for HCMpEF and HCMrEF, respectively), and exclusion of ALDOA did not materially alter this result. These findings suggest that transcript sparsity affects individual low-abundance genes but does not appear to systematically account for the observed transcript-level metabolic pattern (Supplementary Table S1).

This pattern was replicated in single-cell resolution data. In the Chaffin 2022 snRNA-seq dataset (14), pseudobulk analysis of cardiomyocytes demonstrated significant negative enrichment of both Glycolysis (NES = −1.74, FDR < 0.001) and Hypoxia (NES = −1.71, FDR < 0.001) in HCMpEF vs. NF, with similar findings in HCMrEF (Glycolysis NES = −1.55, Hypoxia NES = −1.55; both FDR < 0.01). In the Chaffin cohort, adjustment for sex did not materially alter the pathway-level findings; all six prespecified metabolic pathways retained the same enrichment direction and FDR significance status in both HCMpEF and HCMrEF comparisons, with a maximum absolute change in NES of 0.11. The Liu 2023 snRNA-seq dataset (18) independently confirmed glycolytic transcriptional suppression in HCM (Glycolysis NES = −1.35, FDR < 0.05), but Hypoxia was not (NES = +1.22, FDR = 0.15)—likely reflecting the small sample size (3 controls vs. 10 HCM) and the sparser signal of snRNA-seq in this cohort. The Garmany 2023 bulk RNA-seq dataset (13) further validated Hypoxia negative enrichment (NES = −1.89, FDR < 0.001). Across cohorts, both Glycolysis and Hypoxia gene sets showed predominantly negative enrichment, with Hypoxia reaching statistical significance in multiple independent datasets.

The suppression was not confined to cardiomyocytes. In Chaffin 2022, fibroblasts and macrophages showed similarly downregulated Glycolysis in both pEF (Fib NES = −1.63; Mac NES = −2.02) and rEF (Fib NES = −1.54; Mac NES = −1.90) and Hypoxia pathways (all FDR < 0.01) across both pEF and rEF comparisons. That the same trend appeared in the Liu 2023 dataset as well points to a tissue-wide rather than cell-type-specific metabolic reprogramming in HCM (Figure 2).

**Figure 2:**
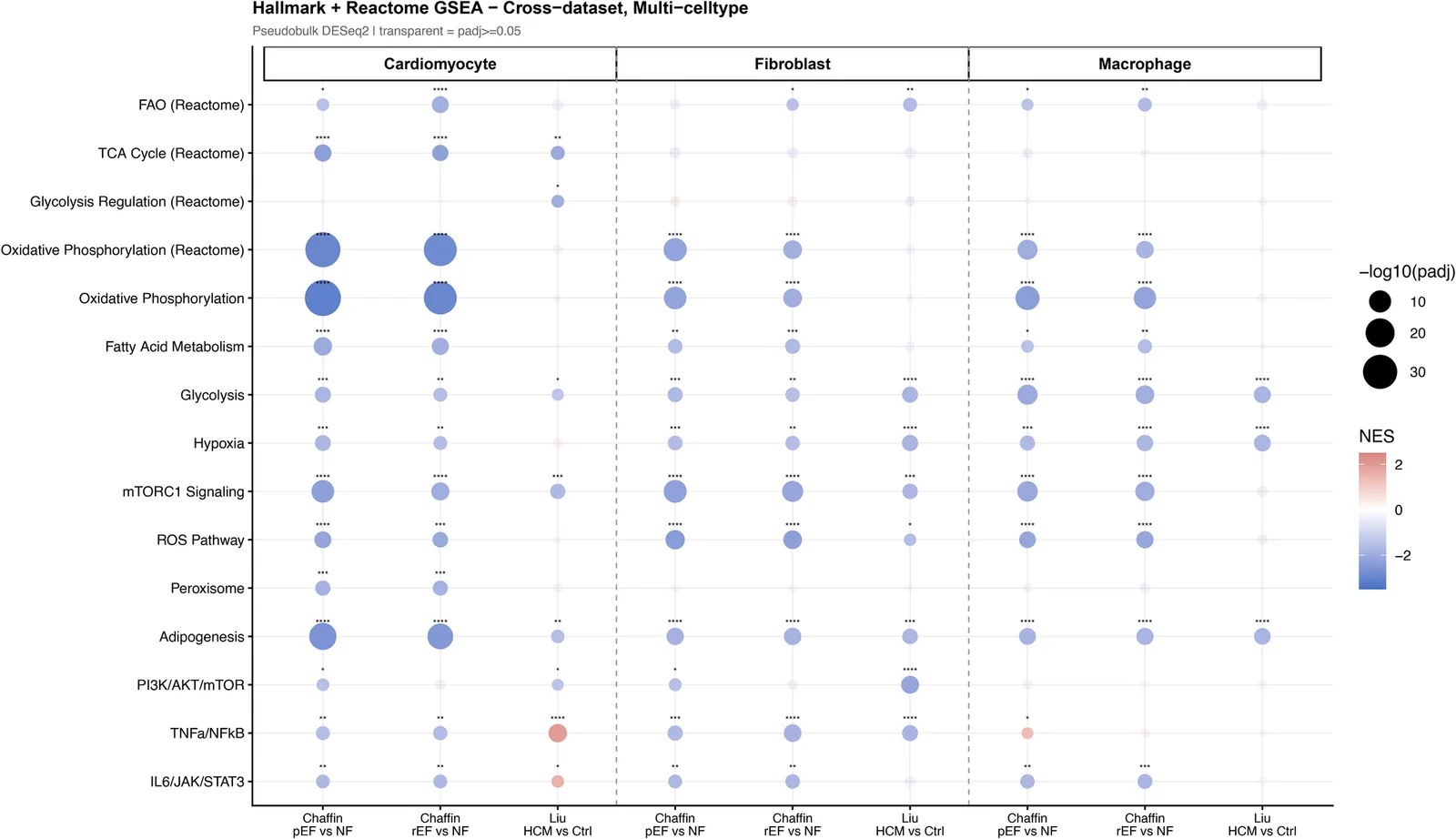
Multi-cell-type Hallmark + Reactome GSEA bubble plot across three cell types and three comparisons. Bubble plot showing GSEA normalized enrichment scores (NES) for selected Hallmark and Reactome metabolic pathways across three cell types (cardiomyocytes, fibroblasts, and macrophages) and three comparisons (Chaffin 2022 HCMpEF vs. NF, Chaffin 2022 HCMrEF vs. NF, and Liu 2023 HCM vs. Control). Bubble color indicates NES (blue = negative enrichment/downregulation; red = positive enrichment/upregulation). Bubble size reflects −log10(*P*adj). Transparent bubbles indicate *P*adj ≥ 0.05. Significance thresholds: *Padj < 0.05, **Padj < 0.01, ***Padj < 0.001, ****Padj < 0.0001. Differential expression was assessed by pseudobulk DESeq2 with Benjamini–Hochberg correction; GSEA was performed using fgsea with 10,000 permutations.

### 3.2 HIF1-related transcriptional output is not increased in human HCM myocardium

Given the consistent downregulation of the HALLMARK_HYPOXIA gene set, we next examined HIF1-associated transcriptional output using three curated target gene sets. In the Garmany 2023 transcriptomic dataset (13), Semenza HIF1 Targets, which is the most established set of direct HIF1α transcriptional targets, showed the strongest negative enrichment of any gene set tested in this study (NES = −1.99, *P*adj = 2.8 × 10^−4^). Elvidge HIF1A Targets (NES = −1.21, *P*adj = 0.25) and PID HIF1A Pathway (NES = −1.02, *P*adj = 0.44) were directionally concordant but did not reach statistical significance (Figure 3).

**Figure 3:**
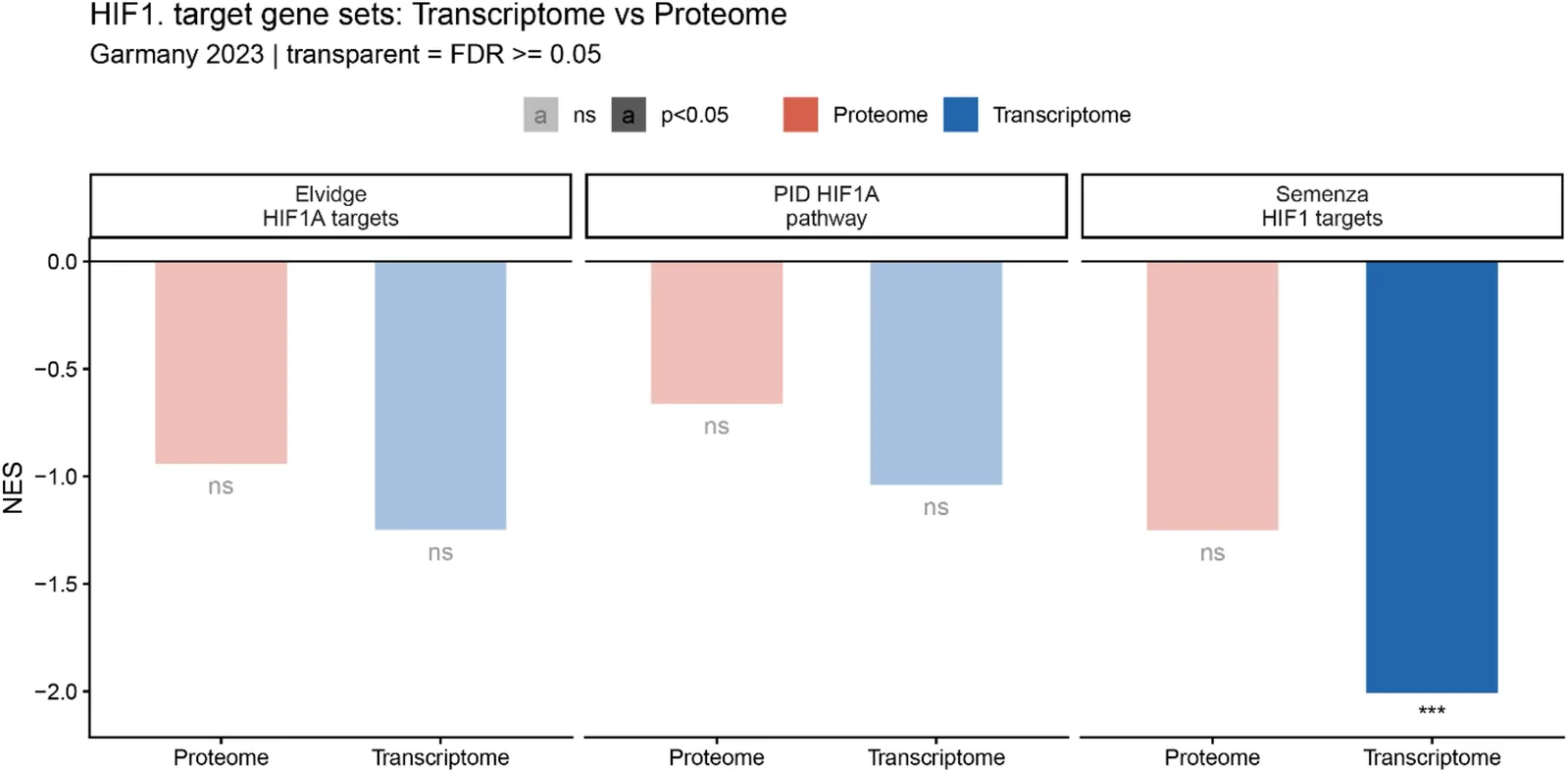
HIF1α-associated transcriptional programs are suppressed at the transcriptomic but not proteomic level in human HCM. GSEA normalized enrichment scores (NES) for three curated HIF1α target gene sets assessed independently in the Garmany 2023 transcriptomic (blue) and proteomic (red) ranked lists. ***Padj < 0.001.

Proteomic GSEA for the same three gene sets trended negative as well, though none passed the FDR threshold—a pattern that foreshadows the broader transcript-protein divergence detailed in the next section. The take-home message is that the Semenza set, which encompasses the best-established direct HIF1α targets (SLC2A1, LDHA, PGK1, ENO1), was strongly suppressed at the transcript level, providing little support for increased HIF1-associated transcriptional programs in human HCM myocardium.

### 3.3 Glycolytic enzymes show transcript–protein discordance in HCM

With the transcriptional picture established, we turned to the protein side, leveraging the Garmany 2023 paired dataset (13), in which RNA-seq and TMT proteomics were performed on the same patient cohort. Among 12 core glycolytic enzymes, 5 showed opposite directions of change between RNA and protein (Figure 4A). Specifically, PFKM, LDHB, and PGK1 were increased at the transcript level but decreased at the protein level, whereas HK1 and PFKL showed the opposite pattern. Overall, the Pearson correlation between RNA and protein log2 fold changes was weak and inverse (*r* = −0.28, *P* = 0.38; *n* = 12). However, given the small number of core glycolytic enzymes, this correlation analysis had limited statistical power and should be considered descriptive rather than inferential. The evidence for discordance therefore rests primarily on the directionally opposing RNA and protein changes observed for individual enzymes.

**Figure 4:**
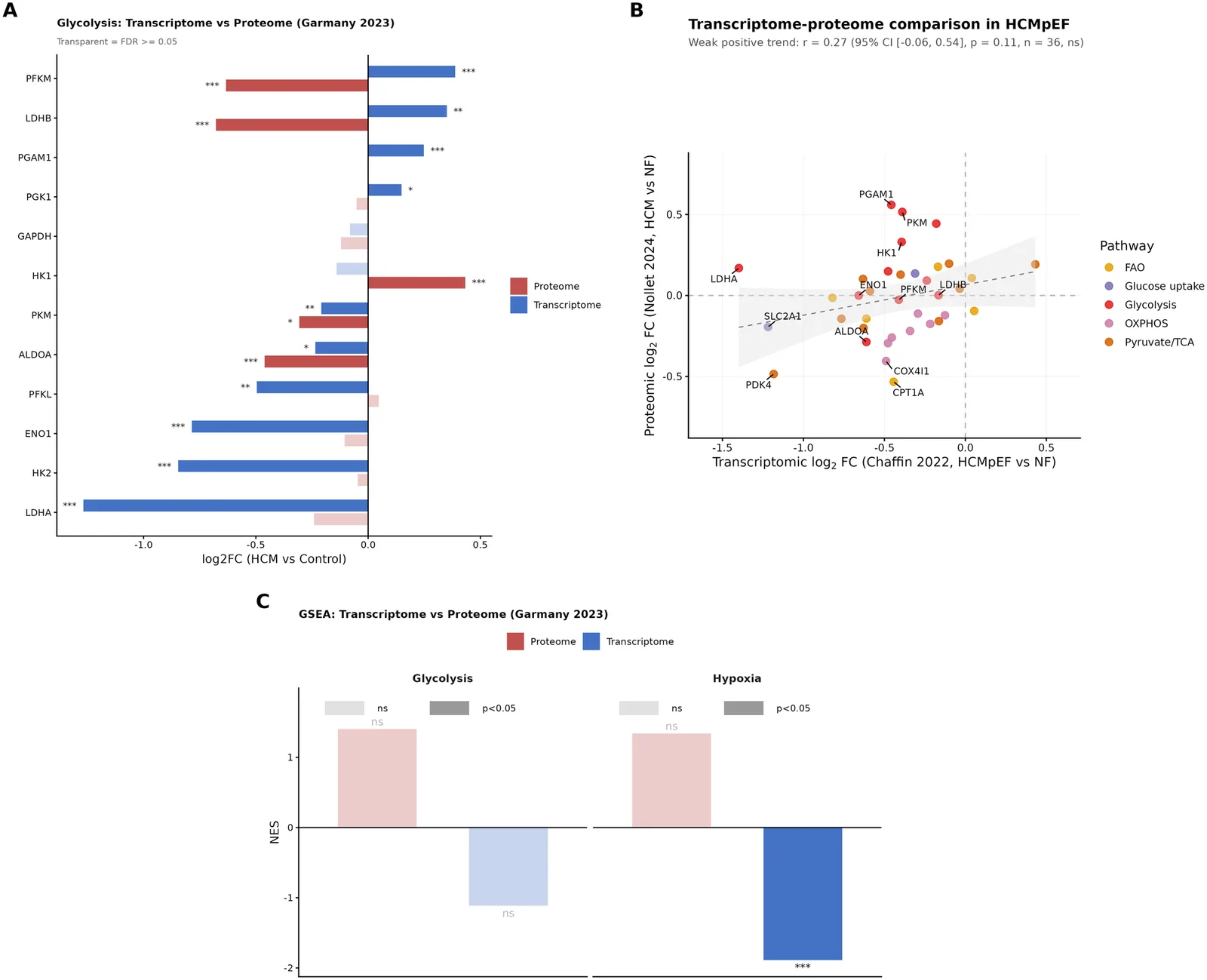
Transcriptome-proteome decoupling in Garmany 2023. **(A)** Paired bar plot showing transcriptomic (blue) and proteomic (red) log2 fold change for 12 core glycolytic enzymes in the Garmany 2023 paired dataset. Transparent bars indicate FDR ≥ 0.05. Significance: *FDR < 0.05, **FDR < 0.01, ***FDR < 0.001. **(B)** Scatter plot of transcriptomic log2FC (HCMpEF vs. NF, Chaffin 2022 snRNA-seq) vs. proteomic log2FC (HCM vs. NF, Nollet 2024 LC-MS/MS) for metabolic genes across five pathway categories. **(C)** Pathway-level GSEA NES comparison for HALLMARK_GLYCOLYSIS and HALLMARK_HYPOXIA between Garmany 2023 transcriptome (blue) and proteome (red) ranked lists. Transparent bars indicate *P*adj ≥ 0.05. ***Padj < 0.001.

At the pathway level, Glycolysis GSEA showed opposite sign at the RNA and protein levels (transcriptome NES = −1.11 vs. proteome NES = +1.40), although neither reached FDR significance (0.348 and 0.080, respectively). HALLMARK_HYPOXIA showed a clearer directional shift, being significantly negatively enriched at the transcript level (NES = −1.89, FDR < 0.001) but positively enriched, though not significantly, at the protein level (NES = +1.34, FDR = 0.155) (Figure 4C). In a supportive cross-dataset comparison, cardiomyocyte-specific transcriptomic changes from Chaffin 2022 (14) were compared with bulk-myocardial proteomic changes from Nollet 2024 (15). PGAM1, PKM, and HK1 showed opposite directions between the cardiomyocyte transcript and bulk-tissue protein levels (*r* = 0.27, Figure 4B). Because these datasets were derived from independent cohorts and the proteomic measurements lacked cell-type specificity, this analysis cannot establish cardiomyocyte-specific transcript–protein discordance and was interpreted only as supportive cross-dataset evidence.

### 3.4 Oxidative pathways show more consistent cross-omics suppression than glycolysis

Additional cross-dataset comparisons supported the possibility that transcript–protein divergence is more prominent in glycolysis than in oxidative metabolic pathways. In the comparison between Chaffin 2022 cardiomyocyte transcriptomic data (14) and Nollet 2024 proteomics (15), PGAM1, PKM, and HK1 showed opposite directions between cardiomyocyte transcriptomic and bulk-tissue proteomic estimates, whereas genes involved in FAO, TCA/pyruvate metabolism, and OXPHOS were more often concordantly reduced (Figure 5A). A similar pattern emerged when comparing Nollet 2024 and Lumish 2024 proteomic datasets: oxidative metabolic proteins were broadly downregulated in both, whereas glycolytic enzymes showed greater cross-platform heterogeneity (Figure 5B). Because the Lumish supplementary table reports only proteins with nominal *P* < 0.05 and provides no protein-level FDR, both datasets are displayed using nominal *P* values; this comparison is therefore descriptive, and the absence of a Lumish data point does not imply the absence of a difference.

**Figure 5:**
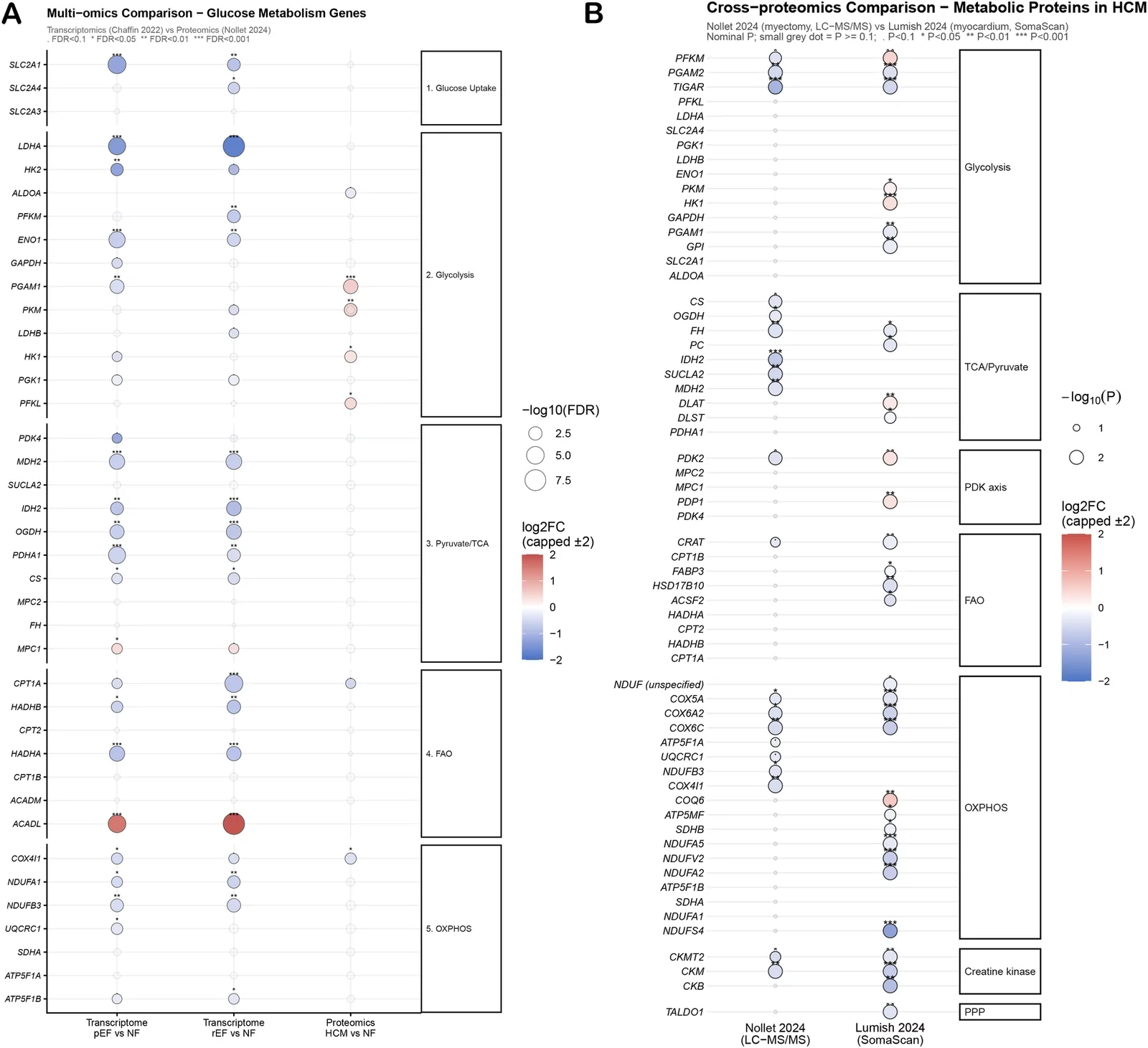
Layered multi-omics comparison of metabolic pathways in HCM. **(A)** Integrated cross-dataset comparison of cardiomyocyte transcriptomic changes from Chaffin 2022 and bulk-tissue proteomic changes from Nollet 2024. Genes are organized into five functional layers: glucose uptake, glycolysis, pyruvate/TCA metabolism, FAO, and OXPHOS. Dot color indicates log2 fold change (capped at ±2), and dot size reflects −log10(FDR). Transparent dots indicate FDR ≥ 0.10. **(B)** Cross-proteomic comparison of metabolic proteins between Nollet 2024 (LC-MS/MS, myectomy tissue) and Lumish 2024 (SomaScan, myocardial tissue). Proteins are grouped by functional category, including glycolysis, TCA/pyruvate metabolism, PDK axis, FAO, OXPHOS, creatine kinase, and the pentose phosphate pathway. Dot color indicates log2 fold change (HCM vs. non-failing; capped at ±2), and dot size reflects −log10 of the nominal *P* value (capped at 2). Both datasets are shown using nominal, unadjusted *P* values as reported in the source supplementary tables. Small grey dots indicate *P* ≥ 0.10; symbols above dots indicate nominal *P* < 0.05 (*), <0.01 (**), and <0.001 (***). Empty positions indicate proteins not detected in, or not listed in the supplementary table of, the corresponding dataset, and do not imply an absence of difference. Lumish protein names were mapped to gene symbols by protein name.

Drilling down into the three-layer glucose metabolism analysis, a hierarchy of disturbance became apparent in cardiomyocytes. At the level of glucose uptake, SLC2A1 (GLUT1) was the most consistently affected gene, showing significant downregulation in both Chaffin cardiomyocyte comparisons and in Liu 2023, whereas SLC2A4 (GLUT4) changed less consistently and SLC2A3 (GLUT3) varied across datasets. Changes in the core glycolytic layer were more uneven. ENO1 showed relatively consistent downregulation, but other glycolytic genes were less uniform across cohorts, with some genes reduced mainly in Chaffin and others showing weaker or inconsistent shifts. By contrast, genes involved in pyruvate oxidation and TCA coupling showed the most coherent pattern of suppression, with CS, IDH2, and OGDH showing the most consistent downregulation, and with PDHA1 and MDH2 also reduced in the Chaffin comparisons. The most reproducible defect, then, is not a wholesale collapse of glycolysis but rather a breakdown in the downstream handoff of glucose-derived carbon to the mitochondrial oxidative machinery (Figure 6).

**Figure 6:**
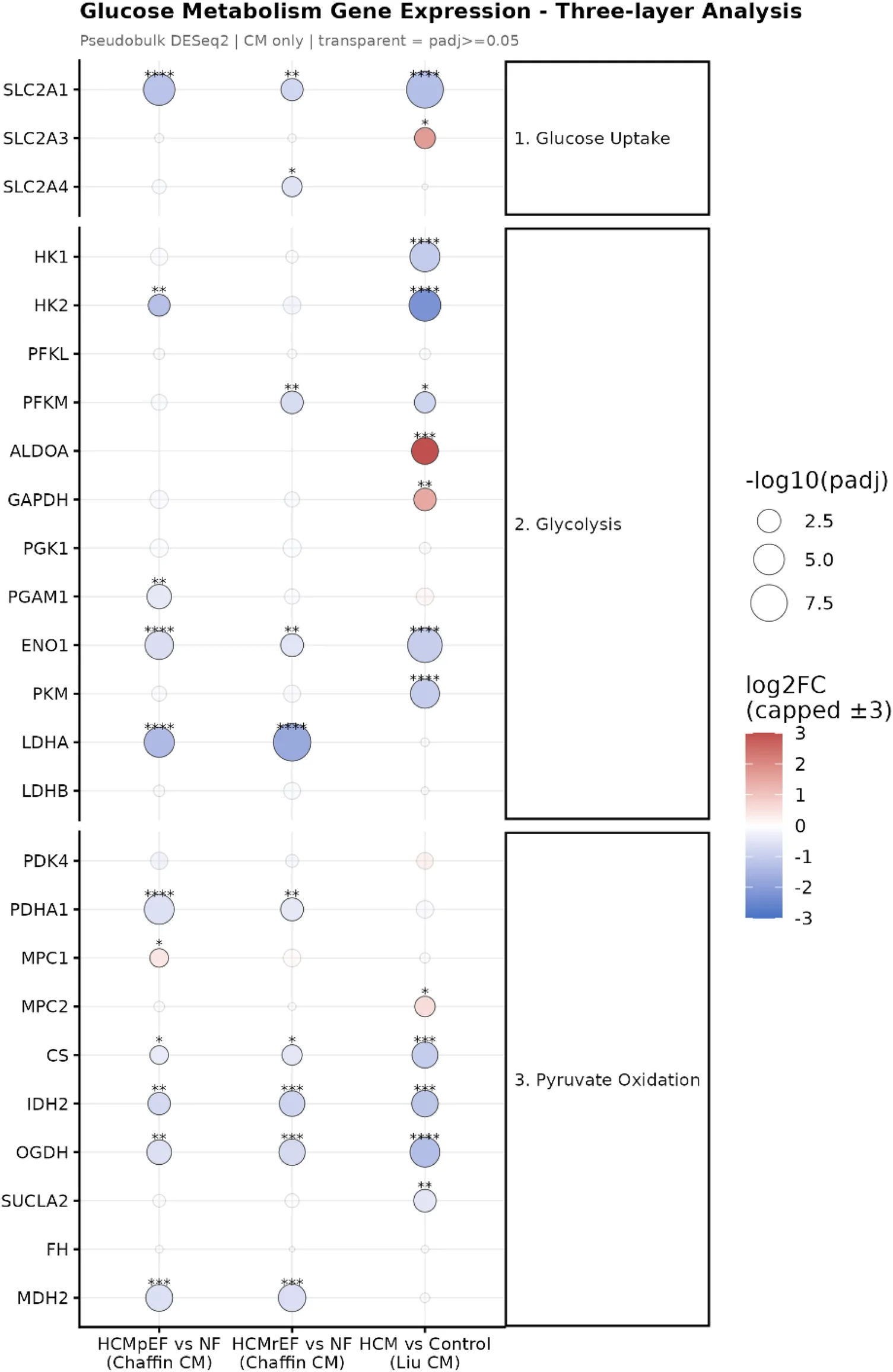
Three-layer analysis of glucose metabolism gene expression in HCM cardiomyocytes. Differential expression of genes involved in glucose uptake, glycolysis, and pyruvate oxidation/TCA coupling is shown across three cardiomyocyte comparisons: HCMpEF vs. non-failing and HCMrEF vs. non-failing in Chaffin 2022, and HCM vs. control in Liu 2023. Dot color represents log2 fold change, dot size corresponds to significance level, and transparent dots indicate non-significant changes.

### 3.5 Phosphorylation changes point to enzyme-specific post-translational regulation

To further examine the basis of the transcriptome–proteome discordance, we compared glycolytic enzymes across transcriptomic, proteomic, and phosphoproteomic layers in the Garmany 2023 dataset (13) using multiple-testing corrected significance thresholds (Figure 7). Glycolytic enzyme phosphorylation showed a split pattern rather than pathway-wide upregulation. Two enzymes, HK1 (phospho log2FC = +0.18, FDR = 5 × 10^−6^) and PGAM1 (+0.40, FDR = 0.02) exhibited significantly increased phosphorylation, with HK1 additionally showing increased protein abundance despite lower transcript levels. Conversely, four enzymes showed significantly decreased phosphorylation (ALDOA: −0.64, FDR = 2 × 10^−5^; LDHB: −0.68, FDR = 7 × 10^−4^; PKM: −0.44, FDR = 0.004; PFKM: −0.71, FDR = 0.008). ENO1 phosphorylation showed a positive trend but did not survive FDR correction (FDR = 0.082). Taken as a whole, the phosphorylation data reinforce the idea that glycolytic remodeling in HCM does not follow a single coordinated program. Each enzyme appears to respond to its own regulatory inputs, producing a mosaic of increases and decreases rather than any uniform compensatory shift.

**Figure 7:**
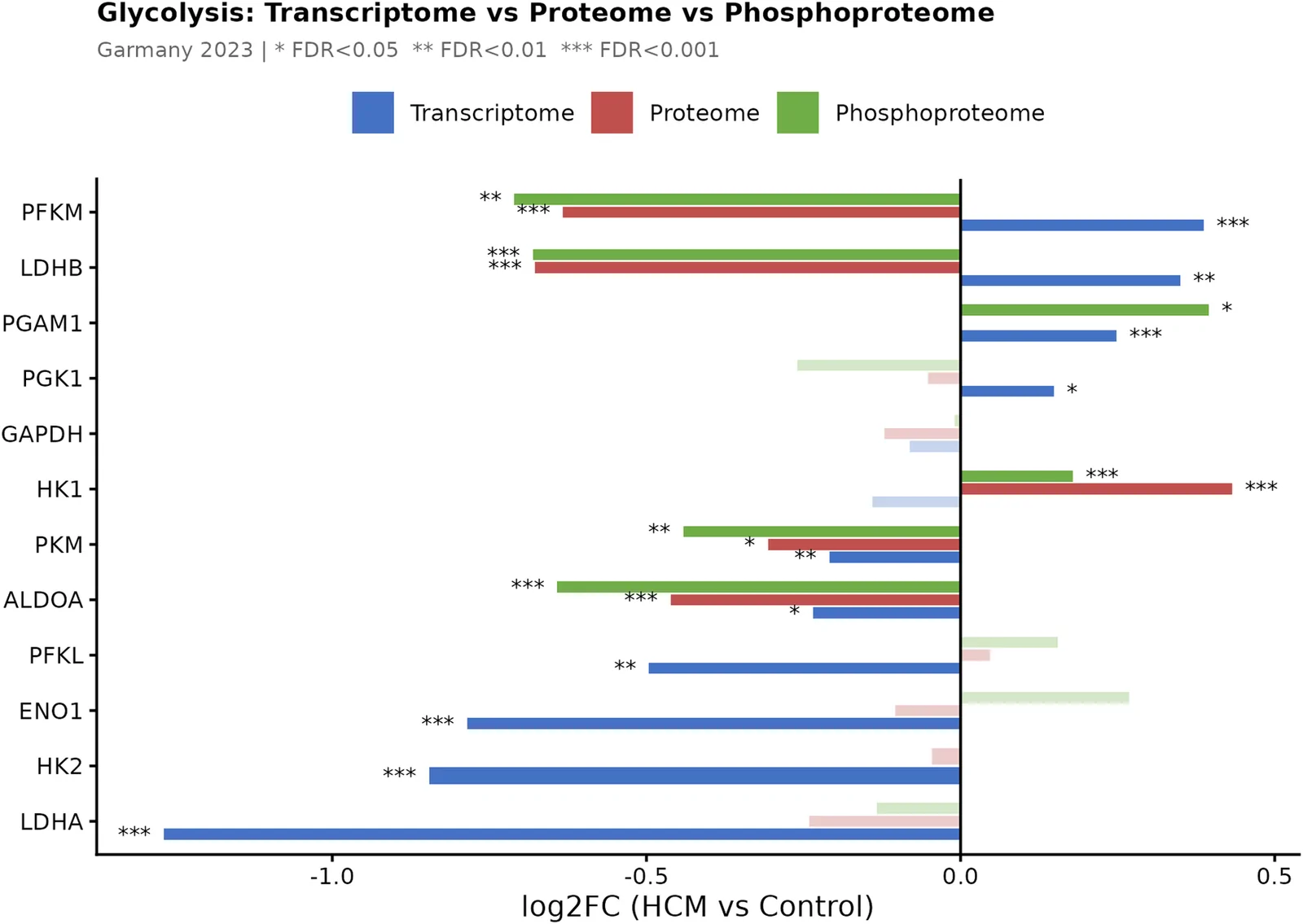
Three-omics integrationof glycolytic enzyme regulation in human HCM. Parallel bar plot showing transcriptomic (blue), proteomic (red), and phosphoproteomic (green) for 12 core glycolytic enzymes in the Garmany 2023 paired dataset (HCM vs. Control). *FDR < 0.05, **FDR < 0.01, ***FDR < 0.001.

### 3.6 Integrated multi-omics analysis supports broad metabolic suppression with limited glycolytic protein compensation

Finally, we brought the three omics layers together in a single integrated view, combining pathway-level GSEA from Chaffin 2022 transcriptomics (14), gene-level protein data from Nollet 2024 (15), and metabolite measurements from the same Nollet 2024 study (15) (Figure 8). At the transcriptomic level (Figure 8A), all major metabolic pathways were negatively enriched in HCMpEF cardiomyocytes, including OXPHOS (NES = −3.12), TCA cycle (NES = −2.31), mTORC1 (NES = −2.30), FAO (NES = −2.03), glycolysis (NES = −1.73), and hypoxia (NES = −1.71), all FDR < 0.001. At the proteomic level (Figure 8B), a divergent pattern emerged: glycolytic enzymes PGAM1, PKM, and HK1 were increased, whereas FAO and OXPHOS proteins (CPT1A, ETFDH), OXPHOS components (COX4I1) and TCA-associated enzymes (IDH2, MDH2) were reduced. At the metabolomic level (Figure 8C), key metabolites consistently supported metabolic suppression, including Acetyl-CoA (log2FC = −1.69, *q* = 0.0004), acylcarnitines C10–C18 (mean log2FC = −1.43, *q* < 0.05), NADH (log2FC = −1.05, *q* = 0.005), and lactate (log2FC = −0.99, *q* = 0.003). That lactate falls even as certain glycolytic enzymes are more abundant is difficult to square with a model of globally elevated glycolytic flux.

**Figure 8:**
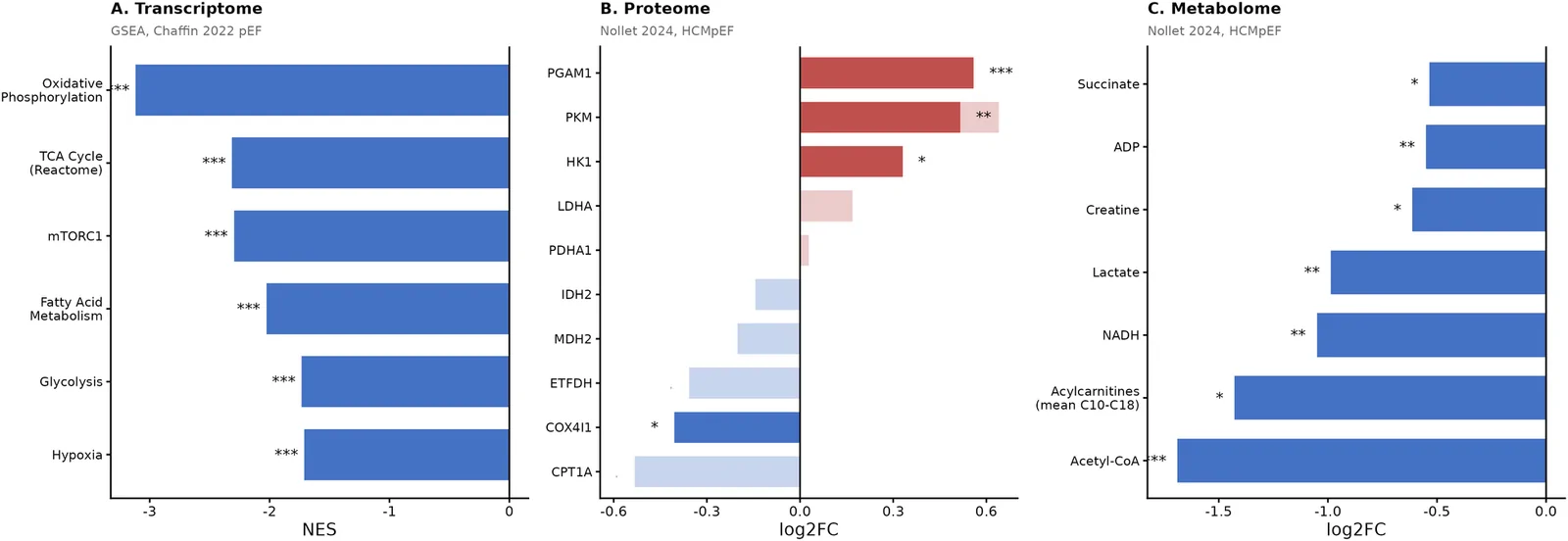
Multi-omics integration confirms metabolic suppression with selective glycolytic protein compensation in human HCM. **(A)** GSEA normalized enrichment scores for six major metabolic pathways in Chaffin 2022 cardiomyocytes (HCMpEF vs. NF). **(B)** Proteomic for selected metabolic enzymes from Nollet 2024 LC-MS/MS (HCMpEF vs. NF). *FDR < 0.05, **FDR < 0.01, ***FDR < 0.001. **(C)** Metabolites from Nollet 2024 targeted metabolomics (HCMpEF vs. NF). *q < 0.05, **q < 0.01, ***q < 0.001.

## 4 Discussion

The central finding of this study—drawn from eight datasets, three omics layers, and over 400 HCM patients—is that glycolysis behaves differently from the rest of the metabolic landscape. Glycolytic transcript programs and HIF1α target genes are suppressed, yet a subset of glycolytic enzymes are maintained or even elevated at the protein level, with phosphorylation changes that vary from one enzyme to the next. Oxidative pathways tell a simpler story: FAO, TCA cycle, and OXPHOS genes go down at both the RNA and protein level in a broadly consistent manner. This selective glycolytic discordance upends the textbook idea of a coordinated “metabolic shift” and sits in tension with recent murine data showing HIF1α activation in sarcomeric HCM (21).

HK1 stood out as the clearest case of this transcript-protein mismatch. Its mRNA was reduced in four out of four transcriptomic cohorts, but its protein was increased across all three proteomic platforms (TMT, LC-MS/MS, and SomaScan)—technologies that work on entirely different principles. On top of that, HK1 phosphorylation was significantly elevated in the Garmany 2023 phosphoproteomic data. The fact that this pattern holds across such methodologically diverse datasets makes it hard to attribute to a technical artifact, and it points to HK1 as a particularly informative target for dissecting post-transcriptional regulation in HCM.

What could explain this disconnect? One possibility is that glycolytic proteins are selectively stabilized—perhaps through AMPK-dependent signaling, which is known to be active in energy-depleted myocardium—so that proteins persist longer even when their transcripts decline (22). A second scenario involves enhanced translation of certain glycolytic mRNAs, something that has been reported in other cardiac stress settings but not yet directly tested in HCM (23). A third possibility involves site-specific post-translational regulation. In the Garmany phosphopeptide data, HK1 Y732 phosphorylation was significantly increased; phosphorylation of this residue has been shown in non-cardiac experimental systems to enhance HK1 catalytic efficiency and glucose affinity (24). However, phosphorylation cannot be interpreted uniformly as enzyme activation. For PGAM1, the well-characterized activating Y26 site was not significantly altered (25), whereas S118 phosphorylation was increased; previous studies have linked PAK1-mediated S23/S118 phosphorylation to reduced PGAM activity (26). These observations emphasize that the functional consequences of phosphorylation are residue-specific, and the present phosphoproteomic data alone do not establish increased glycolytic enzyme activity in HCM myocardium. None of these mechanisms are mutually exclusive, and all remain to be validated experimentally in human HCM cardiomyocytes. The phosphoproteomic data, however, complicate any simple compensatory narrative. HK1 and PGAM1 gained phosphorylation, but ALDOA, LDHB, PKM, and PFKM lost it. If the cell were mounting a coordinated post-translational rescue of glycolysis, one would expect a more uniform phosphorylation increase. The mixed pattern instead points to enzyme-by-enzyme regulation, with each glycolytic step apparently responding to its own set of upstream cues. At the same time, these findings should be interpreted cautiously, since the phosphoproteomic evidence derives from a single dataset and does not directly indicate enzymatic activity or flux.

A second major finding is that HIF1-associated transcriptional output is not elevated and is negatively enriched in several human HCM cohorts. These transcriptomic findings do not directly establish reduced HIF1α protein abundance, stabilization, nuclear localization, or transcription-factor activity. This stands in contrast to recent work by Raj Murthi et al. who showed that HIF1α protein is upregulated in a sarcomeric HCM mouse model and that knocking it out specifically in cardiomyocytes attenuated disease progression (21). How do we reconcile these findings? The most obvious difference is disease stage: human myectomy tissue comes from patients with advanced, symptomatic disease (typically NYHA Class III–IV with severe outflow tract obstruction), while the mouse models capture an earlier, more adaptive phase of hypertrophy. It is plausible that HIF1α is activated transiently during early remodeling and then exhausted or actively suppressed as the disease progresses, a temporal dynamic that cross-sectional human data cannot capture. Species-level differences in metabolic regulation and therapeutic context further limit the direct translatability of murine findings to human pathology.

Metabolomics provides an additional, although indirect, layer of evidence. In the Nollet 2024 dataset (15), myocardial lactate and acetyl-CoA concentrations were reduced in HCM. Because steady-state metabolite abundance does not directly measure pathway flux, these findings cannot establish reduced glycolytic throughput. However, when considered together with the transcriptional suppression of glycolytic and oxidative pathways, they are consistent with altered glucose utilization and impaired coupling of glycolysis to mitochondrial oxidation. One possible interpretation is that constraints may occur at multiple points in glucose metabolism, including glucose uptake and downstream mitochondrial pyruvate oxidation. On the input side, SLC2A1 (GLUT1) transcripts are consistently reduced, potentially limiting glucose entry; on the output side, reduced TCA-cycle and OXPHOS programs may constrain downstream oxidative utilization of pyruvate. The net result is what has been called myocardial “energy starvation”—a state where multiple compensatory mechanisms are engaged but none fully restores energy balance.

A practical lesson from our analysis concerns proteomic methodology. The three platforms agreed on HK1, but disagreed on other enzymes—PGAM1, for example, was up in Nollet 2024 LC-MS/MS (15) and down in Lumish 2024 SomaScan (20), and PFKM showed a similar platform-dependent flip. Mass spectrometry and aptamer-based assays measure protein abundance through fundamentally different chemistries, so some discordance is not surprising, but it does mean that proteomic claims about any single glycolytic enzyme (other than HK1) should not rest on a single platform. Accordingly, candidate metabolic proteins showing platform-dependent effects should be prioritized for orthogonal validation using an independent assay, ideally in the same tissue cohort, and conclusions should place greater weight on changes that are reproducible across analytical platforms than on isolated single-platform findings.

Two broader points follow. First, the absence of increased HIF1-associated transcription in human HCM tissue should prompt caution when extrapolating murine HIF1α findings to clinical settings—the biology of advanced human disease may be qualitatively different from early-stage mouse models. Second, the metabolic profile we observe—reduced lactate, depleted acetyl-CoA, suppressed TCA/OXPHOS—argues that the critical bottleneck is not glycolysis *per se* but the coupling of glycolysis to mitochondrial oxidation. Therapeutically, this would favor strategies that promote pyruvate entry into the TCA cycle (for instance, PDK inhibitors that relieve the brake on pyruvate dehydrogenase) over strategies that simply try to boost glycolytic throughput.

Several limitations related to the source cohorts should be emphasized. Most myocardial samples were obtained from patients undergoing septal myectomy and therefore predominantly represent symptomatic, advanced, obstructive HCM. The molecular patterns identified here may not fully generalize to earlier-stage disease, non-obstructive HCM, or genotype-specific subgroups. In addition, the included cohorts differed in clinical characteristics, disease severity, genetic background, treatment exposure, tissue sampling, and cellular composition. These factors may contribute to cross-cohort heterogeneity and to differences observed across transcriptomic and proteomic platforms. Accordingly, cross-dataset consistency was interpreted as supportive evidence of shared metabolic remodeling, whereas cohort-specific discrepancies were not assumed to reflect biological regulation alone. Moreover, snRNA-seq suffers from transcript dropout, which could disproportionately affect moderately expressed metabolic genes. The proteomic datasets are bulk-tissue measurements and therefore lack cell-type specificity. This is particularly relevant in advanced HCM, where fibrosis and changes in stromal and immune-cell abundance may alter tissue composition. Consequently, bulk protein differences may reflect both changes in protein abundance within individual cell types and shifts in the relative abundance of those cell populations. Thus, comparisons between cardiomyocyte-specific snRNA-seq and bulk proteomics cannot establish cardiomyocyte-specific transcript–protein discordance. We therefore regard the Chaffin–Nollet comparison as supportive cross-dataset evidence, whereas the paired Garmany bulk RNA-seq/proteomic analysis provides the stronger evidence for transcript–protein divergence.

Although donor-level pseudobulk aggregation reduces the impact of single-nucleus sparsity, very-low-detection transcripts remain less precisely estimated. This was particularly evident for ALDOA in the Chaffin dataset, and its individual effect estimate should therefore be interpreted cautiously. However, sensitivity analyses did not identify a systematic relationship between nucleus-level detection and disagreement with independent bulk RNA-seq effect estimates. Finally, the phosphoproteomic evidence rests on a single dataset. Going forward, spatial transcriptomics and single-cell proteomics could resolve cell-type ambiguity, while isotope-tracing experiments (e.g., 13C-glucose flux in iPSC-cardiomyocytes carrying HCM mutations) would directly test whether the glycolytic proteome is indeed functionally constrained, as our data suggest.

## 5 Conclusions

To summarize, our analysis of 8 independent datasets across three molecular layers shows that glycolysis occupies a unique position in HCM metabolic remodeling: its transcriptional programs are suppressed, yet selected enzyme proteins—most consistently HK1—are maintained or increased, with phosphorylation patterns that differ from one enzyme to the next. Oxidative pathways, by contrast, decline in a more coordinated fashion across RNA and protein. The overall picture is one of uneven, pathway-specific regulation rather than a uniform metabolic shift. These findings argue that glycolytic and oxidative branches of cardiac metabolism are governed by distinct regulatory mechanisms in HCM and that future therapeutic and mechanistic work should account for this layer-specific complexity.

## Data Availability

The original contributions presented in the study are included in the article/Supplementary Material, further inquiries can be directed to the corresponding author.
