# Supplementary material for: Selective transcript–protein discordance of glycolytic remodeling in human hypertrophic cardiomyopathy
Source: Front Cardiovasc Med. 2026 Oct 21;13:1937619. doi: 10.3389/fcvm.2026.1937619 (PMC13732749; doi:10.3389/fcvm.2026.1937619)
Supplement: Supplementary file 1 [file Table1.docx]

**Supplementary Table S1.** Quantitative assessment of snRNA-seq detection and comparison with bulk RNA-seq for core glycolytic genes

| **Gene** | **NF median** **detection (%)** | **HCMpEF median** **detection (%)** | **HCMrEF median** **detection (%)** | **Pseudobulk detected donors** **(NF; pEF; rEF)** | **Chaffin pEF** **log2FC** | **Chaffin rEF** **log2FC** | **Garmany bulk** **log2FC** |
| --- | --- | --- | --- | --- | --- | --- | --- |
| **HK1** | 38.4 | 35.1 | 43.7 | 16/16; 8/8; 7/7 | -0.393 | -0.193 | -0.140 |
| **HK2** | 5.0 | 3.0 | 5.5 | 16/16; 8/8; 7/7 | -1.282 | -0.955 | -0.846 |
| **PFKL** | 2.8 | 3.3 | 4.4 | 16/16; 8/8; 7/7 | -0.180 | -0.105 | -0.496 |
| **PFKM** | 31.8 | 29.0 | 31.0 | 16/16; 8/8; 7/7 | -0.411 | -0.710 | +0.387 |
| **ALDOA** | 0.0 | 0.0 | 0.0 | 5/16; 2/8; 3/7 | -0.611 | +0.118 | -0.235 |
| **GAPDH** | 13.4 | 10.2 | 13.1 | 16/16; 8/8; 7/7 | -0.477 | -0.406 | -0.081 |
| **PGK1** | 17.4 | 15.4 | 20.0 | 16/16; 8/8; 7/7 | -0.238 | -0.237 | +0.149 |
| **PGAM1** | 8.5 | 7.9 | 9.6 | 16/16; 8/8; 7/7 | -0.457 | -0.261 | +0.248 |
| **ENO1** | 14.1 | 11.6 | 14.0 | 16/16; 8/8; 7/7 | -0.659 | -0.548 | -0.785 |
| **PKM** | 22.3 | 21.6 | 23.8 | 16/16; 8/8; 7/7 | -0.389 | -0.448 | -0.209 |
| **LDHA** | 9.9 | 4.6 | 4.8 | 16/16; 8/8; 7/7 | -1.399 | -1.767 | -1.268 |
| **LDHB** | 40.5 | 44.2 | 41.0 | 16/16; 8/8; 7/7 | -0.166 | -0.417 | +0.350 |

**Notes.** Detection (%) is the donor-level median proportion of cardiomyocyte nuclei with a Cell Ranger raw count >0 for the indicated gene. Pseudobulk detected donors are shown as NF; HCMpEF; HCMrEF. Chaffin log2FC values are from donor-level cardiomyocyte pseudobulk comparisons (HCMpEF vs NF and HCMrEF vs NF). Garmany bulk log2FC values are from myocardial bulk RNA-seq (HCM vs control). ALDOA was an extreme low-detection outlier in Chaffin cardiomyocytes (10 detected nuclei among 122,362; 0.00817%) and should be interpreted cautiously at the single-gene level.

**Abbreviations:** NF, non-failing; HCMpEF, hypertrophic cardiomyopathy with preserved ejection fraction; HCMrEF, hypertrophic cardiomyopathy with reduced ejection fraction; log2FC, log2 fold change.
